# Mind the Gap: Reporting and Analysis of Sex and Gender in Health Research in Australia, a Cross-Sectional Study

**DOI:** 10.1089/whr.2022.0033

**Published:** 2022-09-12

**Authors:** Lea Merone, Komla Tsey, Darren Russell, Cate Nagle

**Affiliations:** ^1^ College of Healthcare Sciences, James Cook University, Townsville, Queensland, Australia.; ^2^ College of Arts, Society and Education, James Cook University, Smithfield, Queensland, Australia.; ^3^ Cairns Sexual Health Service, Cairns North, Queensland, Australia.

**Keywords:** medical research, sex inclusion, Feminism, health research, sex inequalities

## Abstract

**Introduction:**

Historically, medical studies have underrepresented female participants and most research data have been collected from males and generalized to other genders. This article aims to determine if there is a sex and/or gender gap in recent Australian health research.

**Methods:**

This descriptive cross-sectional study of the published literature examines recent Australian-based clinical trials for inclusion of sex and gender. Medians and interquartile ranges (IQRs) were calculated for study sample sizes and female:male representation. Proportion of sex and/or gender was analyzed by the clinical specialty of the trials. *t*-Tests were used to ascertain significance of any difference in recruitment of female and males.

**Results:**

A total of 88 articles were included in the analysis. Most studies (*n* = 63) were randomized clinical controlled trials. Overall women constituted 55% (IQR 30% of all participants). Of the 71 mixed-sex studies, only 8.9% (*n* = 7) analyzed the data by sex. Women were significantly underrepresented in cardiology and nephrology studies and overrepresented in psychiatry, care of the elderly, and orthopedic studies.

**Conclusions:**

When analyzed by specialty, women are overrepresented in specialties considered to be female patient dominated, such as psychiatry and care of the elderly, and underrepresented in specialties such as cardiology and nephrology. The overrepresentation of women in some specialties can reinforce gender stereotypes, potentially harming women. In addition, exclusion of males from these areas of research may be of disservice to men's health.

## Introduction

Historically, medical studies have shown bias against equitable female inclusion because of concerns that research may harm the female reproductive system or that the variability of menstruation may impact on the reliability of the results.^1,2^ This exclusion of women was further enhanced by perceptions of the male body as the norm.^3^ Consequently, most research data have been collected from cis-gendered males and generalized to females and those who are intersex, transgender, or elsewhere on the gender spectrum^3,4^ (respectfully referred to herein as gender nonbinary). Cis-gendered people are those who identify as the phenotypic gender they were assigned at birth.^5^ There is a call for Australian research policy to align with those in Europe and United States and increase equitability in sex and gender reporting in medical research.^4^

It is recommended that percentages of each sex and gender recruited into clinical research be proportional to the disease-specific sex and gender prevalence.^6^ Sex refers to the biological and physiological characteristics that define humans as female, male, or intersex, while gender is a societal construct that refers to roles, activities, and behaviors, and encompasses a wide range of identities, including woman and man.^4^ Individuals with varying sexes and genders experience diseases and respond to treatments differently and consequentially, clinical research should analyze results by sex and gender.^7^

There are significant discrepancies between the medical care of female and male patients that are likely associated with the deficiency in knowledge of disease manifestation, investigation, and management in females.^8^ Females tend to wait longer than males for a diagnosis or pain relief,^9^ and are more likely to be misdiagnosed or discharged during serious medical events.^10,11^ A recent systematic scoping review of evidence underpinning a sex and gender gap in the Australian and international medical literature highlighted several issues; females remain broadly underrepresented in the medical literature and when females are included adequately, sex and gender are poorly reported and insufficiently analyzed.^12^ The 2007 *National Statement on Ethical Conduct in Human Research* (updated in 2018)^13^ requires fair participant inclusion in Australian clinical trials. However, these guidelines do not specifically recommend sex and/or gender proportions or stipulate performing analysis of data by sex.

The aim of this article is to determine if there remains a sex and/or gender gap in contemporary Australian health research. The objectives of this study were to determine the representation of females in a sample of recently published clinical studies (both randomized controlled trials and observational studies) conducted in Australia, if results are analyzed by sex, and if there is a difference in sex representation between specialties.

## Methods

This descriptive, cross-sectional study of the published peer-reviewed literature examined a sample of the recent (2019) Australian-based clinical studies. The published articles were evaluated for inclusion of sex and the gender spectrum both in the trial and in the analysis of data. This paper utilised publicly available publications and so did not require ethics approval.

Australian studies were defined as those conducted in Australia, utilizing Australian-resident participants. Multicenter trials were included, provided they were conducted in Australia. Age restriction was applied; inclusion of trials was limited to participants 18 years of age or older.

**FIG. 1.**
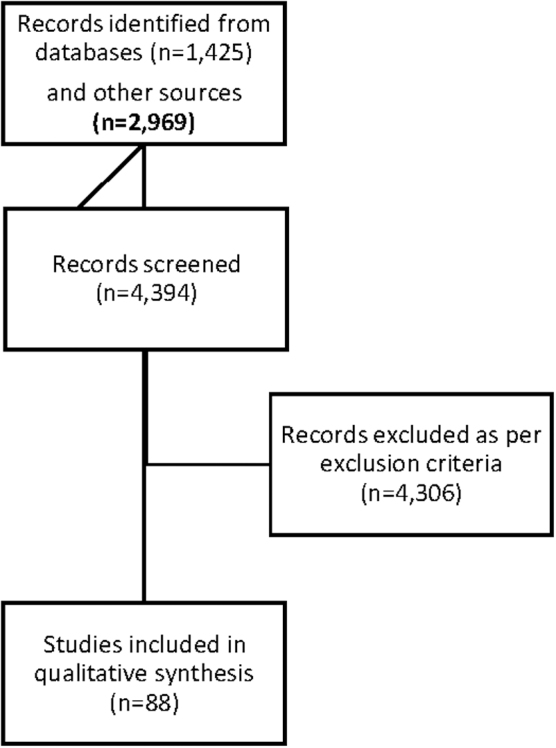
Flow chart of the search strategy and article inclusion/exclusion.

The inclusion and exclusion criteria are provided in Table 1. We excluded clinical trial protocols as there were no results to determine whether data had been analyzed by sex and/or gender. Any text that was not subject to peer review was also excluded (Fig. 1).

**Table 1.** Selection Criteria

Inclusion criteria	Exclusion criteria
Published January 1, 2019 to December 31, 2019	Clinical trial protocol without completed data
RCT or nonrandomized clinical trials, cohort studies, case–control studies, clinical registered trials	Nonpeer-reviewed articles
Hospital or community patient population	
Conducted in Australia	Duplicate article
Adults >/ = 18 years	Full text not available

RCT, randomized controlled trials.

Owing to the large heterogeneity of studies included, we categorized trials by medical specialty, rather than by specific disease. For this reason, it was not possible to determine underrepresentation of females by disease-specific prevalence. We therefore determined that across a specialty, representation of women and men should be 50:50. Medical specialties were determined in one of two ways; if it was stated in the article, it was categorized accordingly, and when the specialty was not stated, the *International Statistical Classification of Diseases and related health problems* (ICD-10) was used.^14^

A search for the terms “Clinical Trial” AND “Australia” AND “adult” was conducted using commonly used databases to obtain a broad sample of articles: PubMed, Medline (hosted by OVID), PsycINFO, and Science Direct (Elsevier). A search of the Australian New Zealand Clinical Trials Register (ANZCTR) was also conducted. Where a trial was listed as completed on the ANZCTR, the article was searched for on the databases listed above. All searches were limited to completed Australian studies published in 2019 as we were exclusively seeking to determine if there is a sex and/or gender gap in contemporary Australian research.

The study was guided by Arksey and O'Malley's^15^ framework with modifications by Levac et al.^16^ Lead author (L.M.) collated studies, removed duplicates, and conducted the initial screen of articles by title and abstract. Full texts of included studies were reviewed independently by two authors (L.M. and K.T.). A data extraction form was developed to tabulate variables of interest: medical specialty, study design, sample size, proportion of females to males, and analysis by sex and/or gender. Where studies gave percentage of females and males by trial arm, an average was calculated. We calculated the median and interquartile range (IQR) for study sample sizes and female:male representation. Proportional sex and/or gender representation were then established by clinical specialty of the trial. Finally, using statistical package Stata14,^17^ we performed paired *t*-tests where possible for representation of females and males across the mixed-sex studies that provided information on sex proportions.

## Results

### Search results

The database search yielded a total of 1425 records and the search of the Australian Clinical Trials Registries^18^ produced 2696 published studies. Following screening of titles and abstracts for selection criteria, a total of 88 articles were included in the analysis.

### Description of sample

Most studies (*n* = 63) were randomized controlled trials (RCTs). Other designs included observational studies (*n* = 19) (of which five were cohort studies) and feasibility/safety or effectiveness trials (*n* = 6).

The 88 studies were divided into mixed-sex (*n* = 71) and single-sex (*n* = 8 female only and *n* = 1 male only) categories. There were eight studies that did not provide information on sex or gender proportions.

The 71 studies with mixed-sex participants included the following specialties: psychiatry (*n* = 22), oncology (*n* = 8), neurology (*n* = 6), gerontology (*n* = 6), pain management (pain) (*n* = 5), cardiology (*n* = 4), respiratory (*n* = 4), orthopedics (*n* = 4), endocrinology (*n* = 2), gastroenterology (*n* = 2), ophthalmology (*n* = 2), nephrology (*n* = 2), general practice (*n* = 1), microbiology (*n* = 1), dermatology (*n* = 1), and complementary medicine (*n* = 1). Of the nine single-sex studies, specialties included were oncology (*n* = 3), obstetrics and gynecology (*n* = 2), sexual health (*n* = 1), gerontology (*n* = 1), rehabilitation (*n* = 1), and psychiatry (*n* = 1).

### Sex and gender representation

There were no studies that explicitly included intersex people or gender nonbinary people. There were eight studies with only female participants, three of which related to gynecological and obstetric health and one relating to each of endocrinology, psychiatry, sexual health, endometrial cancer, and breast cancer. All eight of these studies were specific to female biology, except for the breast cancer study. A single study examining prostate cancer recruited only male participants.

For both the mixed-sex and single-sex studies, the median overall sample size was 107.5 (IQR 291.25), and the range of participants across the studies was 7 to 250,648. The median female sample size across all studies was 55% (IQR 30%), and the median male sample across all studies was 45% (IQR 30%). Of the 79 mixed-sex studies, only 8.9% (*n* = 7) analyzed data by sex.

**FIG. 2.**
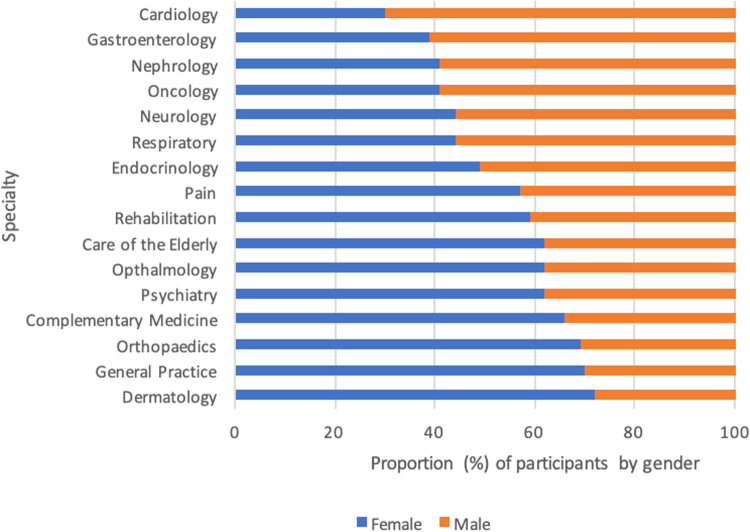
Percentage of females:males included in 2019 Australian studies by specialty.

Representation of sex varied widely between specialties (Fig. 2 and Table 2). Specialties that significantly underrepresented females in Australian research were cardiology (female:male ratio 30:70, *p* = 0.01) and nephrology (female:male ratio 38:62, *p* = 0.02). Specialties that significantly overrepresented females were psychiatry (female:male ratio 67:33, *p* = 0.02), orthopedics (female:male ratio 68:32, *p* = 0.001), and care of the elderly (female:male ratio 65:35, *p* = 0.02).

**Table 2.** Paired *t*-Test Analysis of Sex Representation in Mixed-Sex Studies by Specialty

Medical specialty	Number of studies included	Mean (SD) percentage inclusion of females	Females 95% confidence interval	Mean (SD) percentage inclusion of males	Males 95% confidence interval	*t*-Test score	*p*
Respiratory	4	44.39 (17.88)	15.94 to 72.83	55.61 (17.88)	27.16 to 84.06	−0.63	0.57
Cardiology	4	29.68 (6.88)	18.73 to 40.61	70.32 (6.88)	59.38 to 81.27	−5.91	**0.01**
Psychiatry	22	57.43 (23.04)	47.46 to 67.39	37.79 (21.08)	28.67 to 46.90	2.42	**0.02**
Pain	4	57.1 (8.40)	43.73 to 70.47	42.9 (8.40)	29.53 to 56.27	1.69	0.19
Orthopedics	4	68.90 (3.64)	63.11 to 74.69	31.08 (3.62)	25.31 to 36.83	10.42	**0.00**
Oncology	8	32.91 (21.65)	17.42 to 48.39	57.09 (27.15)	37.67 to 76.51	−2.04	0.07
Ophthalmology	2	61.75 (16.62)	−87.55 to 211.05	38.25 (16.62)	−111.05 to 187.55	1.00	0.5
Neurology	4	46.88 (7.55)	34.85 to 58.90	45.13 (7.55)	41.10 to 65.15	−0.82	0.47
Gastroenterology	2	39 (7.07)	−24.53 to 102.53	61 (7.07)	−2.53 to 124.531	−2.20	0.27
Endocrinology	2	49.35 (34.43)	−260.04 to 358.75	50.65 (34.43)	−258.74 to 360.05	−0.03	0.98
Care of the elderly	5	65.1 (8.92)	54.03 to 76.17	34.9 (8.92)	23.83 to 45.97	3.7	**0.02**
Nephrology	2	41.10 (4.10)	4.25 to 77.95	63.90 (2.97)	37.21 to 90.58	−28.50	**0.02**
Overall	63	48.07 (23.36)	42.83 to 53.30	51.68 (33.64)	36.76 to 46.59	1.69	0.09

Bold text indicates a significant *p* value.

SD, standard deviation.

Of the single-sex studies, three of the eight women-only studies^19–21^ concerned conditions from which biological males do not suffer, such as gestational diabetes and endometrial cancer. One single-sex female study, however, was related to breast cancer,^22^ which affects both females and males. The single male-only study concerned prostate cancer.^23^

There was no clear consensus in the literature on the use of the terms “sex” and “gender.” No study recruited participants outside of the binary sexes female and male, while five studies used the term gender to refer to biological sex.

## Discussion

Our analysis highlights several issues with the diversity of study populations in Australian clinical research. Several studies (*n* = 5) used the term gender to refer to biological sex. Other studies have found that clinical researchers often use the terms sex and gender synonymously and conflating the terms may lead to confusion about the contributions of sex and gender to health and incomplete analysis of health research data.^24^ Krieger argues that greater precision is needed when considering whether to analyze sex and/or gender (or neither) in clinical research, requiring critical thinking and an understanding of how sex and gender interact to determine which is warranted.^25^

### Sex and gender representation in health research

Our study demonstrated that in 2019, females represented approximately half of all participants in Australian health research. This finding is echoed in a large data study in the United States, which also showed a 50:50 female:male representation across all clinical trials, but significant discrepancies between clinical specialties.^8^ Adequate overall female representation across all health research is possibly indicative of the successful and relatively recent efforts to include females in clinical trials, and the development of sex-based therapeutics.^26^

We found that intersex people and gender nonbinary people, however, are severely underrepresented in Australian clinical research, almost to the point of nonexistence outside of the study of sex and gender. Intersex variations include genetic, hormonal, and phenotypic variance; therefore, intersex people, like females, may differ in their presentation of disease and response to treatment when compared to that of the male body. Despite this, other research has shown that there is a significant gap in clinical research outside of the focus on the sexual characteristics of intersex people.^27^ Research suggests that 1.7%–4% of humans have some kind of intersex variation^28^; however, many of these are not apparent without testing, and thus, these estimates may be conservative.^27^ The 2016 Australian census determined there were 1260 sex and/or gender diverse people in Australia and this figure is believed to be an underestimation.^29^

In keeping with our findings, a recent review of the literature (2016) determined far fewer clinical studies of transgender people who explored their health outside of their transition and mental health, and where there were studies surrounding general health, they were exclusively about transgender people rather than inclusion in clinical trials with cis-gendered people.^30^ It has been noted previously that transgender people are “invisible” in clinical research.^31^ Our study demonstrated a total absence in the explicit participation of gender nonbinary people in Australian health research. The exclusion of intersex and gender nonbinary people from clinical research may serve to further disadvantage these population subgroups in much the same way as women, leaving an uncertainty around clinical diagnosis and response to treatment. In addition, once gender nonbinary people are included in health research, subanalysis by sex and gender should be performed.^32^

### Analysis by sex and gender in health research

Analyzing by sex allows researchers to identify differences between females and males, which may be important for clinical diagnosis and management.^25^ Our findings indicate that in 2019, few studies in the Australian clinical literature analyzed their results by sex and none by gender. These findings are in keeping with other studies. DeBruin noted that when women are included in adequate numbers in clinical studies, rarely is sex-specific analysis performed to determine the effects of sex on the results.

When sex analysis is performed, it is often done without regard for advancing women's health, such as how the disease specifically affects women or differences in treatment that may be required.^33^ Analyses of results by sex appear to be poor across all study designs^32–35^ and, as demonstrated by our analysis and other studies, is not improving. According to Vidaver et al., inclusion of women as study subjects did not improve over a 5-year period of analysis following publication of guidelines on the inclusion of female subjects in clinical trials.^36^ More recently, Geller et al. determined that National Institute of Health (NIH) policies have not resulted in significant increases in reporting by sex (or race and ethnicity).^37^

Analysis of results by sex is important; because women's and men's anatomy and physiology are different,^38–41^ females and males may be more prone to different diseases and respond differently to treatments and interventions.^7,41^ The sex and gender research gap may translate into real-life impacts for female patients. For example, organ transplants in female patients are less successful than in male patients,^42^ postmenopausal women respond differently to antidepressant treatment than males,^43^ and females are diagnosed later than males with peanut allergy, despite outnumbering male allergy sufferers.^44^ While subgroup analyses can increase type 1 errors in research,^45^ females have been significantly underrepresented in previous medical research and we maintain it is important to power studies to perform sex-based analysis to aid in closing the research and medical gap between females and males.

### Sex and gender representation in health research by specialty

The results presented in this cross-sectional analysis suggest that women are adequately included in clinical research overall, however, when results were analyzed by clinical specialty, females were shown to be underrepresented in the research of some medical specialties and overrepresented in others. This has been observed in a large data study in the United States, with similar discrepancies noted in cardiology and nephrology research.^8^ This 2021 analysis of sex inclusion in US trials demonstrated that in adult medicine, cardiology clinical trials had the most significant association with lower recruitment of females.^8^

In keeping with this study, our results show that it is possibly the perceptions of sex prevalence^46,47^ that drive recruitment percentages of females into clinical trials, rather than sex prevalence statistics, as recommended by Mastroianni et al. in the *NIH Revitalization Act* (1993).^48^ It is possible that perceptions of disease prevalence are driven by what is observed in clinical practice; however, underrepresentation or overrepresentation of women in health research may alter diagnosis patterns and enhance existing perceptions that may be unfounded.

Studies have reported that the perception of disease and sex biases may be primary drivers for underrepresenting females in medical research, rather than proportion of females and males affected by the disease.^49,50^ Many perceptions of sex-related prevalence appear to be outdated, for example, ischemic heart disease is the leading cause of death in high-income countries in both females and males,^51^ and females experience greater functional disability, symptom burden, and higher prevalence of nonobstructive coronary artery disease than men.^52^ Melloni et al., examined the representation of females in cardiovascular disease randomized controlled trials (RCTs).

Twenty studies recruited only male participants compared to a single study that recruited only female participants. Importantly, female representation was higher in cardiovascular diseases that are perceived to affect females more, such as hypertension and stroke, but lower in diseases perceived to affect fewer females, such as coronary disease.^53^ Welch et al. performed a cross-sectional analysis of Canadian clinical research to ascertain the inclusion of female participants and the extent of sex-based analyses, determining that, while the gap in terms of female inclusion is not large, females are underrepresented in clinical research for some specialties and sex-based analysis is significantly underutilized.^32^

Cardiovascular disease has historically been perceived to be a disease affecting men rather than women.^54–56^ However, evidence shows that in more recent times, this is not the case. In the general adult Australian population in 2018, the absolute risk for lifetime prevalence of heart disease was very similar between women and men with approximately a 1% difference; 4.2% and 5.4%, respectively.^57^ Furthermore, recent statistics from 2019 show that heart disease is increasing in younger women^58,59^ and that outcomes are poorer for women than for men.^60^ This may be partially due to the observed research gap.

Females with acute myocardial infarction present with different symptoms than men, are less likely to have their infarction identified during diagnostic angiography than men,^61,62^ and are often underinvestigated and consequentially less likely to be managed correctly.^60^ The rising incidence of cardiovascular disease in young females younger than 40 years^63^ does not appear to correspond to an increase in representation in the recent research literature. The Australian Longitudinal Study on Women's Health^64^ is helping to decrease the research gaps; however, as our analysis highlights, in general cardiology research studies, females remain underrepresented.

In high-income countries, the prevalence of chronic kidney disease is higher in women than in men,^65^ yet this is not reflected in the proportion of females recruited to nephrology research. Renal physiology is also different in females and males, females have 12% fewer glomeruli than males^66^ and female sex hormones increase the synthesis of angiotensinogen, but decrease synthesis of angiotensin-converting enzyme, both of which increase the risk of hypertension.^67^ Furthermore, in the general adult population, the lower muscle mass of females compared to males means that reference ranges for estimates of glomerular filtration rate in blood tests may be inappropriate.^67^ Despite these important differences, sex differences are neglected in nephrology research.^68^ In keeping with our findings, analysis of female participation in research by burden of disease in the United States demonstrated that, alongside oncology, neurology, and immunology, nephrology had the lowest female enrolment relative to disability-adjusted life years.^8^

Our study suggests that research in the care of the elderly appears to be biased toward females; however, this may be owing to the longer lifespan of women; there are more elderly women than men and consequentially are more likely to present to services and be recruited into trials. Despite their longer lifespan, women of all ages are more likely than men to report poor health and see their doctor; therefore, in this age group, where women often outnumber men,^69^ it is possible that there may be some selection bias when recruiting to clinical trials.

Research conducted in Canada also suggests that women report poorer health because of their longer lives and therefore greater likelihood of developing health problems.^69^ It is possible that this factor is also at play when considering the results for orthopedic studies, which included predominantly elderly patients older than 60 years; osteoporosis is related to falling estrogen levels and more common in elderly females.^70^

The findings from this study suggest that research in psychiatry appears to also be significantly dominated by female participants, even though high numbers of both women and men are affected by psychiatric ailments. Of note, men are more likely than women to die by suicide, whereas women remain more likely to be diagnosed with an anxiety or personality disorder.^71^ The dominance of female participants in psychiatry is possibly rooted in historical perceptions of women as “hysterical.”^72^ Hysteria was the first mental health disorder that was considered a “female disease,” driven historically by perceptions of women as “weak” and “guilty” of sins or supernatural influence.^73^

As a diagnosis, hysteria diminished in the 1970s and was deleted from the *Diagnostic and Statistical Manual of Mental Disorders III* (DSM III) in 1980,^73^ although it has been argued that other terms such as anxiety, borderline personality disorder, and bipolar mood disorder have come to replace this label for female patients, and these conditions have consequentially become stigmatized and potentially overdiagnosed.^74^ If this is the case, it may assist in the explanation of the sex and gender gap swung in the favor of the female participant in psychiatric research. We postulate that this may then become a self-fulfilling prophecy and enhance perceptions of women as anxious or hysterical, to the detriment of women's health. Mental ill-health in women is frequently misunderstood, potentially leading to bias and prejudice.^74^

### Limitations

Many of the included studies used the terms sex and gender interchangeably. This lack of definition and clarity is a limitation of this study, and to mitigate this, we explored sex and/or gender representation in the published health research literature. The data collected were study results published during a single year and therefore may not be truly representative of clinical trials across Australia. The data collected were also limited to that which was published and it is possible that some of the studies analyzed results by sex and found no difference, and therefore did not include these results in their publication.

Heterogeneity across studies made analysis by disease unfeasible; therefore, we analyzed data more broadly by specialty, and assumed that the required representation of female and male participants be 50:50. This assumption was used as the basis for determining overrepresentation and underrepresentation. Finally, on subspecialty analysis, some sample sizes were small and therefore may not be an accurate representation of female inclusion.

Restricting studies to those conducted in Australia, and that utilized Australian-resident participants limited the inclusion of studies in our analysis, but by focusing on research in Australia, our findings can inform Australian public health.

## Conclusion and Implications for Public Health

Overall, the inclusion of women in clinical research conducted in Australia appears balanced; however, when analyzed by specialty, women are overrepresented in specialties perceived to be female patient dominated, such as psychiatry, and significantly underrepresented in specialties such as cardiology and nephrology. Despite increasing numbers of female participants in medical research, there is observable overrecruitment and underrecruitment of females in some clinical specialties. Overrepresentation of women in some specialties can reinforce gender stereotypes, potentially harming women. In addition, exclusion of males from these areas of research may be a disservice to men's health. Where women are adequately represented, there is rarely gender-based analysis, and we believe this should be a requirement for publication in journals. Intersex and gender nonbinary people also remain underrepresented in general medical research.

## Abbreviations Used

DSM III: *Diagnostic and Statistical Manual of Mental Disorders III*
ICD: *International Statistical Classification of Diseases*
IQR: interquartile range
RCT: randomized controlled trial
SD: standard deviation
